# Thermal Modeling and Calibration Method in Complex Temperature Field for Single-Axis Rotational Inertial Navigation System

**DOI:** 10.3390/s20020384

**Published:** 2020-01-09

**Authors:** Zihui Wang, Xianghong Cheng, Jingjing Du

**Affiliations:** 1 School of Instrument Science and Engineering, Southeast University, Nanjing 210096, China; 230179254@seu.edu.cn; 2 Key Laboratory of Micro-Inertial Instrument and Advanced Navigation Technology, Ministry of Education, Southeast University, Nanjing 210096, China; 3 Chinese Academy of Aerospace Science and Engineering, Guiyang 550009, China; Dujingjing7054@126.com

**Keywords:** single-axis rotational inertial navigation system, inertial measurement units, thermal calibration, multiple regression method, BP neural network

## Abstract

Single-axis rotational inertial navigation systems (single-axis RINSs) are widely used in high-accuracy navigation because of their ability to restrain the horizontal axis errors of the inertial measurement unit (IMU). The IMU errors, especially the biases, should be constant during each rotation cycle that is to be modulated and restrained. However, the temperature field, consisting of the environment temperature and the power heating of single-axis RINS, affects the IMU performance and changes the biases over time. To improve the precision of single-axis RINS, the change of IMU biases caused by the temperature should be calibrated accurately. The traditional thermal calibration model consists of the temperature and temperature change rate, which does not reflect the complex temperature field of single-axis RINS. This paper proposed a multiple regression method with a temperature gradient in the model, and in order to describe the complex temperature field thoroughly, a BP neural network method is proposed with consideration of the coupled items of the temperature variables. Experiments show that the proposed methods outperform the traditional calibration method. The navigation accuracy of single-axis RINS can be improved by up to 47.41% in lab conditions and 65.11% in the moving vehicle experiment, respectively.

## 1. Introduction

Inertial Navigation System (INS), as an entirely self-contained system, can obtain attitude, velocity and position of the vehicle by resolving the data sampled by its Inertial Measurement Unit (IMU) containing tri-axis gyroscopes and accelerometers [1]. With the characteristics of high reliability and data rate, INS is widely used in airplanes, missiles, automobiles, submarines, ships, and robots [2,3,4,5]. However, the navigation errors of INS are mainly caused by the gyroscope drifts and accelerometer biases. In order to improve the accuracy of INS without using the high-cost and high-precision IMU, the Rotational Inertial Navigation System (RINS) has been proposed [6,7,8]. In a RINS, the IMU is mounted on a rotary table and forced to rotate along the given axes back and forth to modulate the errors of IMU from constant to periodically varying components, so as to reduce the navigation errors [9].

The research on RINS can date back to 1968 when Geller proposed a local level inertial platform that continuously rotated in azimuth and concluded that the platform rotation attenuated the system position error [10]. From then on, RINS based on IMU of various accuracies have been widely studied. In 1980s, with the development of laser gyroscopes, RINSs based on laser gyroscopes were used for marine navigation, with the ability to supply accurate navigation information for several days [11,12]. In 2004, Yang and Miao analyzed the single-axis RINS based on the fiber-optic gyroscopes [13]. In 2013, Sun et al. proposed a MEMS-based RINS in which the significant sensor biases were compensated to attenuate the navigation errors [14].

RINS can be divided into single-axis [8,15], dual-axis [16,17] and tri-axis [18,19] according to the number of rotation axis. Theoretically, at least two rotation axes should be used to reduce the impact of all tri-axis IMU errors. However, the errors of horizontal IMU contribute more to the navigation system [20]. Hence, the single-axis RINS with its low cost and characteristic of restraining the IMU errors in horizontal axis has been widely used for high-accuracy navigation. Although the rotation modulation can fulfill self-compensation of IMU errors during the alignment and navigation process, the premise is that the IMU errors especially the biases are constant during each rotation cycle. However, the environment temperature and the power heating during the single-axis RINS cold starts affect the IMU performance and make the IMU biases change over time, which cannot be modulated by rotation and will decrease the accuracy of single-axis RINS.

In order to compensate the IMU errors caused by the temperature, plenty of works have been done according to the grade of IMUs, and it has been proved that the thermal calibration process is time-consuming and costly. There are currently two main approaches for thermal calibration: The Soak method and the Ramp method. The Soak method works on the premise of stable sensor temperature while the Ramp method is based on time-varying sensor temperature. Although time-consuming, the Soak method can achieve better accuracy. In 2013, Niu et al. proposed a fast thermal calibration method for low-grade IMU based on the Ramp method and concluded that the compensation accuracy based on the proposed method was close to the Soak method and the calibration time was significantly reduced [21]. In 2016, Wang et al. proposed a Soak method to calibrate the MEMS IMU’s biases, scale factor errors and non-orthogonalities varying with temperature [22]. In 2017, Zhang et al. proposed a parameter-interpolation method to calibrate the MEMS gyroscope’s biases and scale factor errors caused by the temperature [23]. In 2018, Yang et al. utilized the calibration data in 10 temperature points based on the Lagrange interpolation method to calibrate the tri-axial MEMS gyroscope over a full temperature range [24]. With the development of artificial intelligence, many thermal calibration methods for IMU based on Back Propagation neural network [25], Elman neural network [26] and fuzzy neural network [27] have been proposed, and it is concluded that the compensation results are more accurate than the traditional thermal calibration method.

**Figure 1:**
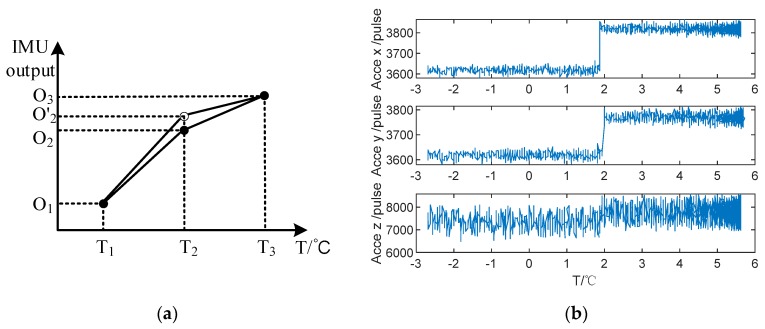
The sectional compensation error analysis. (**a**) The schematic of sectional compensation. (**b**) The accelerometers output at segment point.

In a word, although the thermal calibration method for IMU has been widely researched, some problems are still not settled. Firstly, the thermal calibration model lacks the reflection for the work environment. There exists a temperature field caused by the power of single-axis RINS heating and the environmental temperature change. What is more, the temperature field is more complicated during the cold start process. Besides, both polynomial and interpolation calibration methods utilize the sectional compensation method among all temperature points. However, the segment points may change because the IMU biases are different due to the repetition priming as shown in Figure 1a, and it makes the compensated IMU data step-like at the segment points as shown in Figure 1b, which decrease the position accuracy of single-axis RINS.

Hence, in order to reflect the temperature field change accurately, especially during the cold start process, the sensor temperature, temperature change rate and temperature gradient inside and outside the sensor should be introduced to the thermal calibration model. Besides, an overall compensation method should be proposed to overcome the drawbacks of the sectional compensation method.

The main purpose of this paper is to improve the accuracy of single-axis RINS over a work temperature range by thermal modeling and calibration of IMU. The rest of this paper is organized as follows. The single-axis RINS error analysis with the IMU biases is made in Section 2. The effects of the temperature on the change of IMU biases are analyzed based on the experiment data in Section 3. The thermal calibration method based on the multiple nonlinear regression method and BP-neural network method are proposed in Section 4. The analysis of simulation and experiment results are presented in Section 5. Finally, the conclusions are concluded in Section 6.

## 2. Single-Axis RINS Error Analysis

### 2.1. Definition of Frames

The frames used in the paper are listed in Table 1.

**Figure 2:**
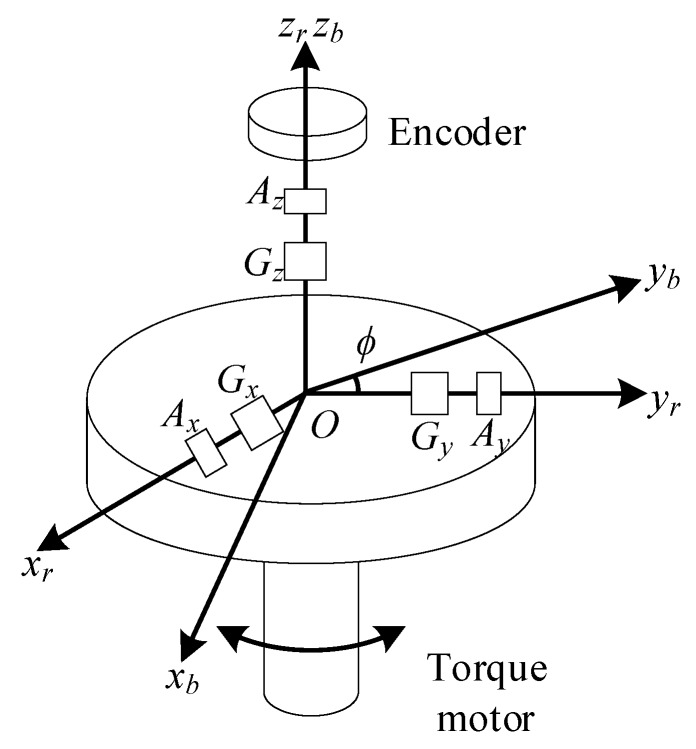
The structure of single-axis RINS.

The system structure of single-axis RINS is shown in Figure 2. The single-axis RINS mainly includes IMU, which is composed of a tri-axis gyroscope and a tri-axis accelerometer, a rotation framework, an angular encoder, and a torque motor. The torque motor drives IMU to rotate periodically along the vertical body axis, and the angular encoder provides rotation angle relative to the body frame. The *r*-frame refers to the rotation frame, which varies with the change of IMU pointing direction in real time. The *b*-frame is defined as the rotation axis *z_s_* that overlaps with the *z_b_*. The relationship between two frames is defined as the rotation angle *ϕ*, and the *r*-frame coincides with the *b*-frame completely when *ϕ* is zero. The rotation modulation of the system is the classical four-position modulation (−135°, 45°, 135°, −45°) used in many RINS [28].

### 2.2. The Single-Axis RINS Error Analysis with the IMU Biases

The errors restraint principle of single-axis RINS can be explained by the following analysis. At time *t*, the direction cosine matrix from *r*-frame to *b*-frame can be described as follows:

(1) $$C_{r}^{b}\left(t\right)=\left[\begin{array}{ccc} \cos\omega_{r}t & -\sin\omega_{r}t & 0\\ \sin\omega_{r}t & \cos\omega_{r}t & 0\\ 0 & 0 & 1 \end{array}\right]$$

where *ω_r_* is the rotation speed, and *ϕ* = *ω_r_* × *t*.

The angular rate and specific force output of gyroscopes and accelerometers in *r*-frame are

(2) $${\tilde{\omega }}_{ir}^{r}={\left(C_{r}^{b}\right)}^{T}\omega_{ib}^{b}+\delta \omega_{ib}^{r}+\omega_{br}^{r}$$

(3) $${\tilde{f}}_{ir}^{r}={\left(C_{r}^{b}\right)}^{T}f_{ib}^{b}+\delta f_{ib}^{r}+f_{br}^{r}$$

where ${\tilde{\omega }}_{ir}^{r}$, $\omega_{ib}^{b}$, ${\tilde{f}}_{ir}^{r}$, and $f_{ib}^{b}$ are the raw output and the ideal output of gyroscopes and accelerometers respectively; *δ*[·] denotes the error of the vector [·]; $\omega_{br}^{r}$ and $f_{br}^{r}$ are the rotation rate and the specific force between *b*-frame and *r*-frame in *r*-frame.

The angular rate and specific force output of gyroscopes and accelerometers in *b*-frame are

(4) $${\tilde{\omega }}_{ib}^{b}=C_{r}^{b}{\tilde{\omega }}_{ir}^{r}+\omega_{rb}^{b}$$

(5) $${\tilde{f}}_{ib}^{b}=C_{r}^{b}{\tilde{f}}_{ir}^{r}+f_{rb}^{b}$$

where ${\tilde{\omega }}_{ib}^{b}$, ${\tilde{\omega }}_{ir}^{r}$, ${\tilde{f}}_{ib}^{b}$, and ${\tilde{f}}_{ir}^{r}$ are the raw output of gyroscopes and accelerometers in *b*-frame and *r*-frame respectively. $\omega_{rb}^{b}$ and $f_{rb}^{b}$ are the rotation rate and the specific force between *r*-frame and *b*-frame in *b*-frame.

For the sake of simplicity, we only consider the biases as the IMU errors and ignore the scale factor errors and misalignment errors of IMU. Take (1), (2) and (3) in (4) and (5) respectively, and we have:

(6) $${\tilde{\omega }}_{ib}^{b}=\omega_{ib}^{b}+C_{r}^{b}\left(\delta \omega_{ir}^{r}+\delta \omega_{rb}^{r}\right)\;=\;\omega_{ib}^{b}+\left[\begin{array}{c} \varepsilon_{x}\cos\omega_{r}t-\varepsilon_{y}\sin\omega_{r}t\\ \varepsilon_{x}\sin\omega_{r}t+\varepsilon_{y}\cos\omega_{r}t\\ \varepsilon_{z} \end{array}\right]$$

(7) $${\tilde{f}}_{ib}^{b}=f_{ib}^{b}+C_{r}^{b}\left(\delta f_{ir}^{r}+\delta f_{rb}^{r}\right)\;=\;f_{ib}^{b}+\left[\begin{array}{c} \nabla_{x}\cos\omega_{r}t-\nabla_{y}\sin\omega_{r}t\\ \nabla_{x}\sin\omega_{r}t+\nabla_{y}\cos\omega_{r}t\\ \nabla_{z} \end{array}\right]$$

where $\delta \omega_{ir}^{r}=\varepsilon ={\left[\varepsilon_{x},\varepsilon_{y},\varepsilon_{z}\right]}^{T}$ and $\delta f_{ir}^{r}=\nabla ={\left[\nabla_{x},\nabla_{y},\nabla_{z}\right]}^{T}$ are the gyroscopes drifts and accelerometers biases respectively. Besides, the rotation error of the torque motor is ignored, which means that $\delta \omega_{rb}^{r}=\;\delta f_{rb}^{r}=\;0$.

Apparently, by rotating the IMU about the vertical axis, the gyroscope drifts and accelerometer biases in horizontal axes are modulated into periodic signals, whose average value is zero in a rotation period *T* = 2π/*ω_r_*. For the sake of simplicity, we assume *b*-frame is aligned with *n*-frame, which means $C_{n}^{b}$ is an identity matrix, then the horizontal attitude errors and velocity errors can be calculated as:

(8) $$\left[\begin{array}{c} \delta \varphi_{E}\\ \delta \varphi_{N} \end{array}\right]=\left[\begin{array}{c} \int_{t=0}^{t=T}\left(\varepsilon_{x}\cos\omega_{r}t-\varepsilon_{y}\sin\omega_{r}t\right)dt\\ \int_{t=0}^{t=T}\left(\varepsilon_{x}\sin\omega_{r}t+\varepsilon_{y}\cos\omega_{r}t\right)dt \end{array}\right]$$

(9) $$\left[\begin{array}{c} \delta V_{E}\\ \delta V_{N} \end{array}\right]=\left[\begin{array}{c} \int_{t=0}^{t=T}\left(\nabla_{x}\cos\omega_{r}t-\nabla_{y}\sin\omega_{r}t\right)dt\\ \int_{t=0}^{t=T}\left(\nabla_{x}\sin\omega_{r}t+\nabla_{y}\cos\omega_{r}t\right)dt \end{array}\right]$$

Hence, the horizontal IMU biases are self-compensated in a complete rotation cycle based on the premise that the IMU biases are constant during each rotation cycle. However, the environment temperature and the power heating when the single-axis RINS cold starts affect the IMU performance and make the IMU biases change over time. As shown in Equations (8) and (9), if the IMU biases change in a rotation cycle, the effects of modulation for single-axis RINS will decrease and the navigation errors may accumulate and grow rapidly. Hence, the IMU biases caused by the temperature should be compensated in order to improve the position accuracy of single-axis RINS.

## 3. The Effects of Temperature on IMU Biases

In order to analyze the effects of temperature on IMU biases, there are several equipments: the two-axis turntable with a thermal chamber, and the temperature sensors inside and outside the IMU. The performance of thermal calibration equipment is shown in Table 2. The thermal chamber is used to simulate the temperature change of the work environment. It should be pointed out that the IMU chip is packaged with the temperature sensor inside, and attached is a temperature sensor in the surface of the package, which is defined as the outside temperature sensor. The inside temperature sensor reflects the sensor heating, while the outside temperature sensor reflects the power heating and the environmental temperature change.

For the high-precision navigation grade IMU, the soak method is more accurate than the ramp method. Hence, we utilize the soak method to analysis the temperature field when the single-axis RINS cold starts at different temperature points. The single-axis RINS was installed on the turntable in the quasi-static state, and the thermal chamber was set at seven temperature points, which is shown in Table 3. These temperature points have covered the working temperature range of single-axis RINS. The single-axis RINS kept thermal insulation for 3 h before the cold start, and then turned on the single-axis RINS without the torque motor rotation at each temperature point.

**Figure 3:**
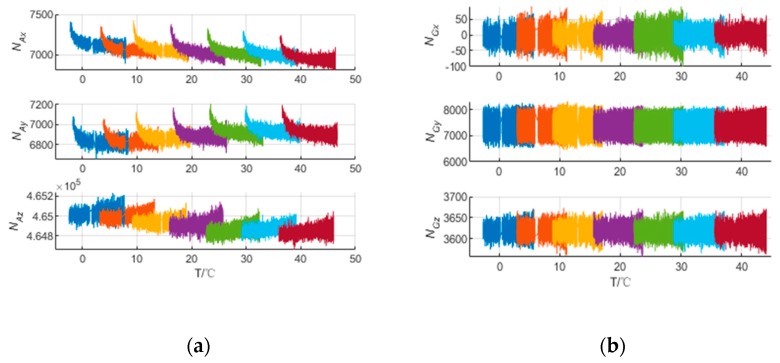
The IMU output at different temperature points. (**a**) The accelerometers outputs. (**b**) The gyroscopes outputs.

The IMU output at different temperature points is shown in Figure 3. It should be pointed out that the outliers caused by the torque motor vibration were excluded. Obviously, the IMU biases change variously at different temperature points, and the accelerometers of single-axis RINS are more sensitive to temperature field than the gyroscopes. In order to ensure the effects of modulation for single-axis RINS, the IMU biases caused by temperature should be calibrated and compensated. The traditional calibration methods based on the polynomial or interpolation method utilize the sectional calibration and compensation method at different temperature points. However, it results in the IMU output errors shown in Figure 1b and decreases the position accuracy of single-axis RINS. Besides, the temperature gradient is ignored in the traditional method, hence, the temperature variation between the environment and the single-axis RINS cannot be reflected in the IMU thermal model.

## 4. Multiple Regression Method and BP Neural Network

### 4.1. The Multiple Regression Method

In order to reflect the temperature field of single-axis RINS, the inner temperature of IMU, the temperature change rate of IMU, and the temperature gradient between the sensor inside and outside are considered in the temperature model of IMU. To overcome the drawback of sectional compensation, the temperature model of accelerometers and gyroscopes proposed in this paper is as follows:

(10) $$N^{\prime }=N-f\left(T,\dot{T},\Delta T\right)$$

where ***N*** and ***N’*** are the IMU output before and after compensation, respectively. T, $\dot{T}$, and ΔT are the inner temperature, temperature change rate and temperature gradient between the inside and outside IMU, respectively. The biases of IMU caused by the temperature can be summarized as:

(11) $$f\left(T,\dot{T},\Delta T\right)=\mathrm{diag}\left(\left[\begin{array}{l} a_{0x}a_{T1x}\cdots a_{Tpx}a_{\dot{T}1x}\cdots a_{\dot{T}qx}a_{\Delta T1x}\cdots a_{\Delta Tsx}\\ a_{0y}a_{T1y}\cdots a_{Tpy}a_{\dot{T}1y}\cdots a_{\dot{T}qy}a_{\Delta T1y}\cdots a_{\Delta Tsy}\\ a_{0z}a_{T1z}\cdots a_{Tpz}a_{\dot{T}1z}\cdots a_{\dot{T}qz}a_{\Delta T1z}\cdots a_{\Delta Tsz} \end{array}\right]\left[\begin{array}{ccc} 1 & 1 & 1\\ T_{x} & T_{y} & T_{z}\\ \vdots & \vdots & \vdots \\ T_{x}^{p} & T_{y}^{p} & T_{z}^{p}\\ {\dot{T}}_{x} & {\dot{T}}_{y} & {\dot{T}}_{z}\\ \vdots & \vdots & \vdots \\ {\dot{T}}_{x}^{q} & {\dot{T}}_{y}^{q} & {\dot{T}}_{z}^{q}\\ \Delta T_{x} & \Delta T_{y} & \Delta T_{z}\\ \vdots & \vdots & \vdots \\ {\left(\Delta T_{x}\right)}^{s} & {\left(\Delta T_{y}\right)}^{s} & {\left(\Delta T_{z}\right)}^{s} \end{array}\right]\right)$$

where $a_{0i}$ (*i* = *x*, *y*, *z*) are the constant biases; $a_{T1i}$ … $a_{Tpi}$ (*i* = *x*, *y*, *z*) are the temperature coefficients; $a_{\dot{T}1i}$ … $a_{\dot{T}qi}$ (*i* = *x*, *y*, *z*) are the temperature change rate coefficients; $a_{\Delta T1i}$ … $a_{\Delta Tsi}$ (*i* = *x*, *y*, *z*) are the temperature gradient coefficients; and *p*, *q*, *s* are the order of the temperature variables.

The purpose of thermal calibration based on the multiple regression method proposed in this paper is to estimate the temperature coefficients in Equation (10). The thermal calibration data were collected as shown in Figure 3, and the temperature coefficients can be calculated using the multiple regression method. Compared to the traditional method, the IMU output does not show a step-like pattern caused by the sectional compensation, which is not acceptable for high-precision single-axis RINS. Besides, the temperature gradient is considered in the model to fully describe the temperature field.

### 4.2. BP Neural Network

**Figure 4:**
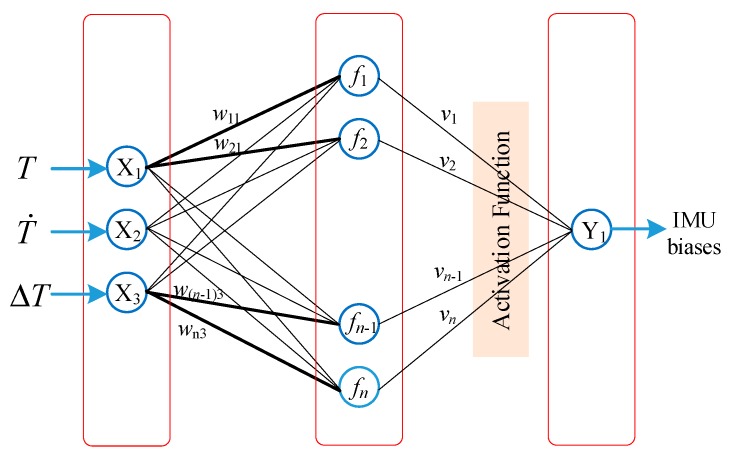
The BP network topology.

The multiple regression method utilizes the polynomial of temperature variables to establish the temperature model. However, the relationship between the temperature variables is more complicated, and there are coupled items among the temperature, temperature change rate and temperature gradient of IMU. Hence, BP neural network is introduced to solve the problem. The BP neural network is an algorithm for error back propagation training with a multi-layer network structure. The connection weight coefficients and thresholds are adjusted by the back-propagation of the output error, hence the error of the network is minimized to achieve the desired precision. The topology used in this paper is shown in Figure 4.

It is a three-layer network structure, consisting of an input layer, a hidden layer, and an output layer. The input layer consists of three neurons, and the relationships among the three layers can be characterized by the following formulas:

(12) $$f_{n}=\sum_{i=1}^{n}w_{ni}\cdot X_{i}+b_{i}$$

(13) $$Y=f_{active}\left(\sum_{i=1}^{n}v_{i}\cdot f_{i}+b^{\prime }\right)$$

where *n* is a neuron in the hidden layer, and *i* is the number of input layers; *w_ni_*, *v_i_*, *b_i_*, and *b*’ are the weight coefficients and biases between the input layer, the hidden layer and the output layer respectively; *f_active_*() is the activation function using the sigmoid function for the output of hidden layer.

(14) $$f_{active}\left(x\right)=\frac{1}{1+e^{-x}}$$

The output neuron is the IMU biases caused by temperature, and the output layer and the hidden layer are connected by an activation function. The IMU biases are known when the single-axis RINS is installed on the turntable, hence the training data can be collected and used to train the BP network.

## 5. Thermal Calibration and Verification Experiments

In this section, some experiments in the lab and field condition are designed to verify the two proposed thermal calibration methods in practice. The lab navigation experiments and moving vehicle experiments are carried out based on the single-axis RINS. The single-axis RINS consists of a tri-axis fiber optic gyroscope, and tri-axis quartz accelerometer with precisions of 0.01°/h and 0.01 mg respectively. Firstly, the thermal calibration was performed based on the traditional method and the two proposed methods, then the IMU calibration was done based on our previous work [29] to transform the pulse output into the rotation rate and specific force.

### 5.1. Analysis of Thermal Calibtation and Cold Start Experiments

The thermal calibration experiments are conducted using three methods:

(1) The traditional thermal calibration method based on the polynomial sectional method.
(2) The multiple regression method descibed in Section 4.1.
(3) The BP neural netwok proposed in Section 4.2.

**Figure 5:**
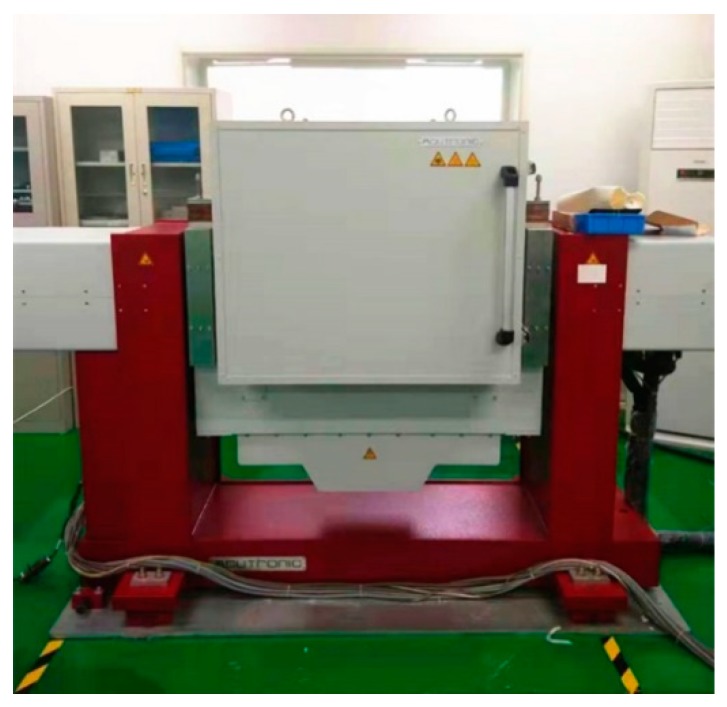
The two-axis turntable with a thermal chamber.

**Figure 6:**
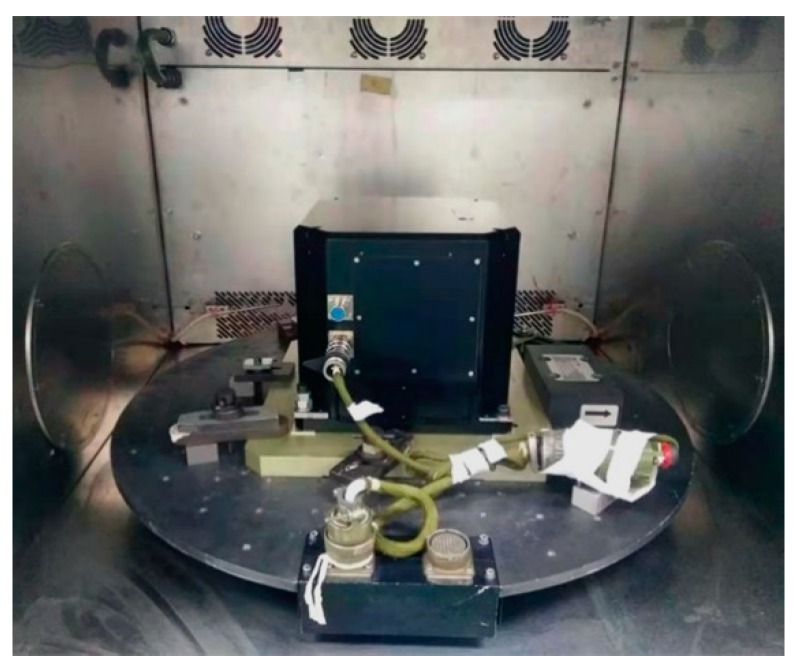
The single-axis RINS installed on the turntable.

The experiments are performed on the two-axis turntable with a thermal chamber as shown in Figure 5, and the performance of the thermal calibration equipment is shown in Table 2. The single-axis RINS are installed on the turntable in the quasi-static state shown in Figure 6.

The thermal chamber is set at seven temperature points as shown in Table 3. The single-axis RINS maintains thermal insulation for 3 h before the cold start; when the temperature field is stable, the single-axis RINS is turned on without the torque motor rotation at each temperature point. The IMU raw outputs are collected and used to calibrate the IMU biases caused by temperature based on those three methods.

#### 5.1.1. The Thermal Calibration Experiments Based on Method 1

In method 1, the thermal model is a piecewise function:

(15) $$N^{\prime }=\{\begin{array}{cc} N-f_{1}\left(T,\dot{T}\right) & T\leq T_{1}\\ N-f_{2}\left(T,\dot{T}\right) & T_{1}<T\leq T_{2}\\ \vdots & \vdots \\ N-f_{6}\left(T,\dot{T}\right) & T_{5}<T\leq T_{6}\\ N-f_{7}\left(T,\dot{T}\right) & T\geq T_{6} \end{array}$$

where *T_i_* (*i* = 1, 2, …, 6) are the sectional temperature points. The thermal model in method 1 consists of the temperature and temperature change rate but ignores the temperature gradient between the work environment and single-axis RINS. The calibration results of *x*-axis IMU based on method 1 are shown in Table 4. There are 7 × 7 = 49 coefficients for the accelerometer and 3 × 7 = 21 coefficients for the gyroscope in the piecewise function model.

**Figure 7:**
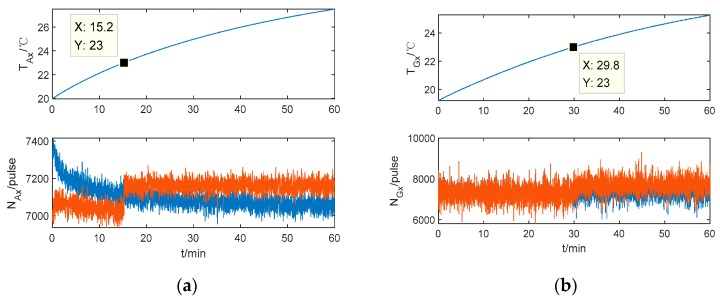
The *x*-axis IMU outputs based on method (1). (**a**) The *x*-axis accelerometer output. (**b**) The *x*-axis gyroscope output.

In order to verify the compensation effects in segment points, the experiment of repetition priming is performed when the temperature of the thermal chamber is set at 25 °C. The x-axis IMU outputs before and after compensation are shown in Figure 7. It is shown that: Firstly, the output of IMU appears as a step-like pattern at the segment temperature point 23 °C, because the IMU biases change in repeat starts and the constant thermal coefficients *A*_0_ and *G*_0_ are not appropriate for the new data at the segment points.

Secondly, the *x*-axis accelerometer output fluctuates at the beginning of the cold start, which reflects that the calibration model based on method 1 should be developed. The temperature and temperature change rate in the thermal model of method 1 are inadequate to reflect the temperature field of single-axis RINS.

#### 5.1.2. The Thermal Calibration Experiments Based on Method 2

The Root Mean Square (RMS) is introduced in order to evaluate the calibration error, and it is defined as

(16) $$RMS=\sqrt{\frac{\sum_{i=1}^{n}\left({\hat{Y}}_{i}-Y_{i}\right)}{n}}$$

where *n* denotes the sample number of IMU output; ${\hat{Y}}_{i}$ denotes the estimation of IMU output, while $Y_{i}$ denotes the IMU output.

In method 2, the relationship between the order of the temperature variables in the thermal model and the RMS of the calibration error is shown in Table 5. The order of, T, $\dot{T}$, and ΔT are *p*, *q*, and *s* respectively as shown in Equation (10).

Table 4 shows that: Firstly, the RMS of calibration error decreases while the order of temperature variables increases. However, the differences between the 3rd order model and 4th order model for accelerometers are small. Secondly, the RMS of error for gyroscopes are almost unchanged, which means that the thermal model with *p* = 1, *q* = 1 and *s* = 1 is fit for the gyroscopes. Hence, the thermal models are set: *p* = 3, *q* = 3, *s* = 3 for the accelerometers and *p* = 1, *q* = 1, *s* = 1 for the gyroscopes. The calibration results based on method 2 are shown in Table 6.

**Figure 8:**
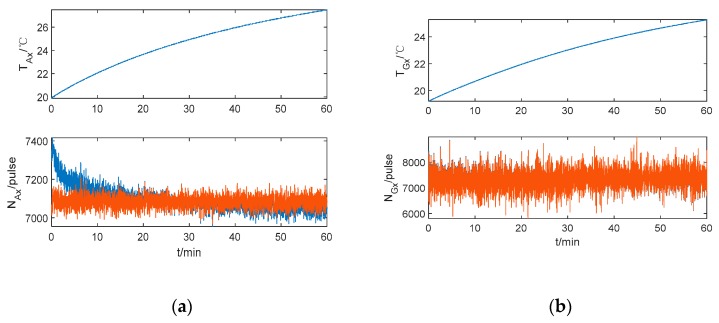
The *x*-axis IMU outputs based on method (2). (**a**) The *x*-axis accelerometer output. (**b**) The *x*-axis gyroscope output.

There are only 10 thermal parameters for the accelerometer and four thermal parameters for the gyroscope. The thermal model of method 2 is more concise compared to method 1. The IMU outputs before and after compensation based on method 2 are shown in Figure 8. It can be seen that the biases of IMU caused by the temperature can be compensated well. In addition, in the repetition priming experiment, the IMU outputs are continued without step-like appearance and there is no fluctuation for the accelerometer at the beginning of the cold start. Besides, the IMU output is nearly constant with the temperature change during the cold start, which agrees with the premise that the IMU biases do not change during each rotation cycle. Hence, the position error caused by the IMU biases can be modulated as shown in Equations (8) and (9), and the effects of modulation lead to restraining the accumulation of position error caused by the IMU biases.

#### 5.1.3. The Thermal Calibration Experiments Based on Method 3

In method 3, 70% of the IMU raw outputs shown in Figure 3 are utilized to train the BP neural network and 30% for validation and testing. In order to choose the number of neurons in the hidden layer, the simulations are performed, and the relationships between the number of neurons and the RMS of calibration error are shown in Table 7. It shows that compared with Table 6, the RMS of the calibration error for accelerometers decrease, while there is little change for gyroscopes because the gyroscope’s outputs are less sensitive to the temperature field. Besides, the RMS of calibration error decreases while the number of neurons increases, but it is not changed basically when *n* ≥ 6 for accelerometers and *n* ≥ 2 for gyroscopes. It is well known that the more neurons there are, the more computational complexity there is for BP neural networks. Hence, considering both accuracy and computational complexity, the number of neurons of hidden layer is set as *n* = 6 and *n* = 2 for accelerometers and gyroscopes, respectively.

The calibration results for x-axis IMU based on method 3 are shown in Table 8. There are 31 coefficients for the accelerometer and 11 coefficients for the gyroscope. Although the coefficients are more than those in method 2, it is shown that the thresholds and weight coefficients between the three layers connect the temperature variables together and introduce the coupled items into the thermal calibration model. Hence, the RMS of calibration error based on method 3 is smaller than that based on method 2, which means that the IMU output is more approximate to constant and the IMU biases can be modulated into a period signal in order to decrease the position error of single-axis RINS.

### 5.2. Analysis of Thermal Verification Experiment

In order to verify the proposed thermal calibration method, the navigation experiments of single-axis RINS in lab and the vehicle experiments are performed.

#### 5.2.1. Navigation Experiments in Lab

**Figure 9:**
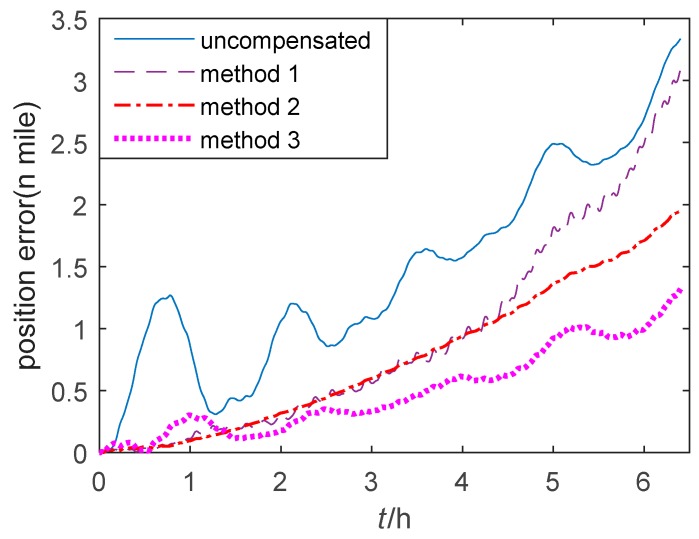
The position error of single-axis RINS in the thermal verification experiment.

In this step, the navigation experiment is performed in the two-axis turntable with the thermal chamber at first. The temperature of the thermal chamber is set from −2 °C to 50 °C with the temperature rate of 0.2 °C/min. The position errors of single-axis RINS in the quasi-static state based on three methods are shown in Figure 9. It is shown that the position error based on method 1 is smaller than the uncompensated error, however, it may increase in some segment points. Compared with method 1, the position error based on method 2 is more steady and does not increase at the segment points. Besides, the position error based on method 3 is the smallest among the three methods. It is verified that the proposed methods (method 2, 3) improved the position accuracy of single-axis RINS in a large temperature range.

**Figure 10:**
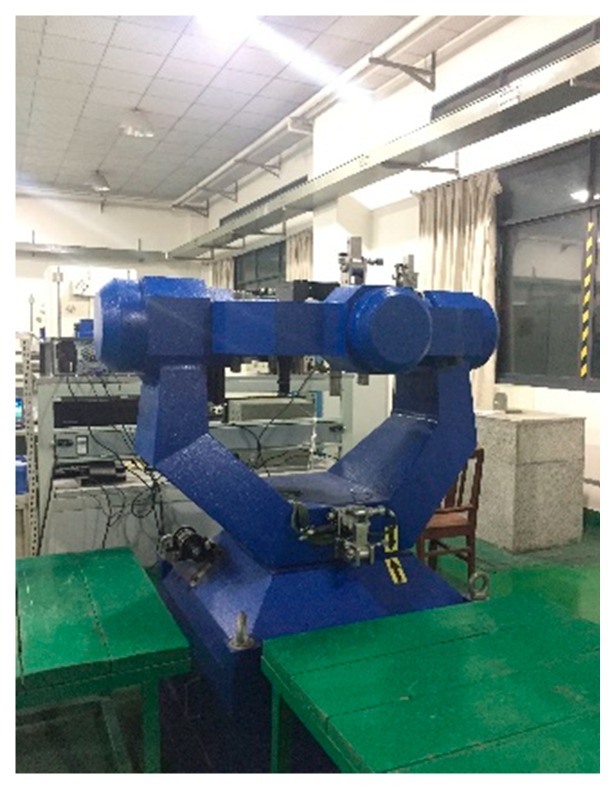
The single-axis RINS installed on the three-axis turntable.

In order to verify the position accuracy based on three thermal calibration methods during the cold start, the self-alignment and navigation experiments are performed in lab. The single-axis RINS is installed on the three-axis turntable shown in Figure 10, which offers the rotation rates and angles of single-axis RINS as reference. The single-axis RINS cold starts in the quasi-static state and the swing state as shown in Table 9, where Heading, Pitch and Roll denote the rotation axis of the turntable.

**Figure 11:**
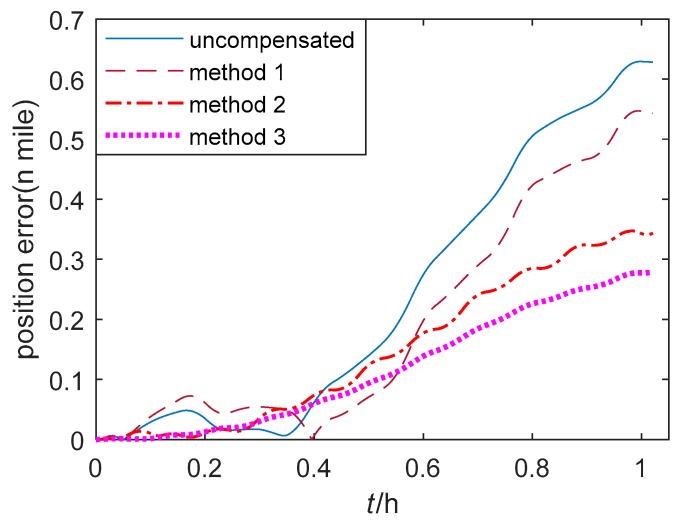
The position error of single-axis RINS in the quasi-static state.

**Figure 12:**
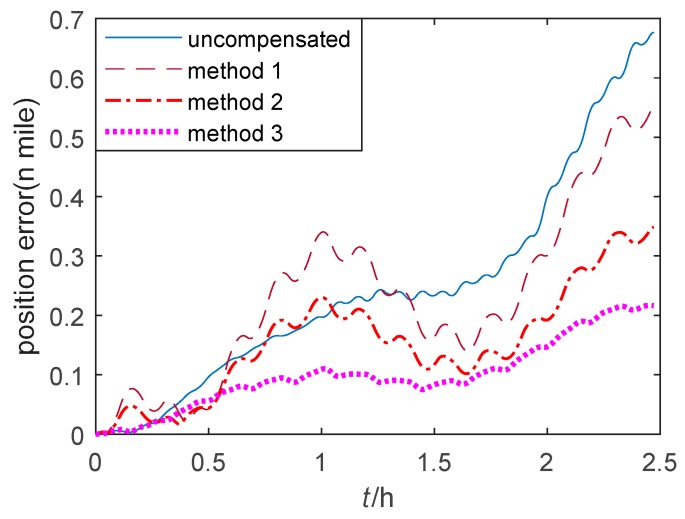
The position error of single-axis RINS in the swing state.

The position errors of single-axis RINS based on three thermal calibration methods in the quasi-static state and the swing state are shown in Figure 11 and Figure 12 respectively. Method 1–3 are described in Section 5.1. It is obvious that the position errors of method 1 fluctuate at the beginning of the cold start, and they are larger compared to those position errors of the proposed method 2. Besides, the position errors of method 3 are the smallest and the most steady among the three methods.

**Figure 13:**
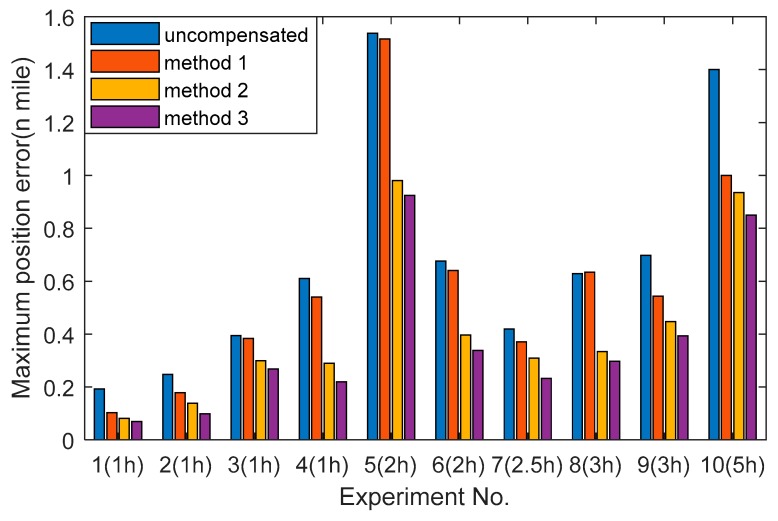
The position errors and time of multiple navigation experiments.

Other navigation experiments in the lab condition are performed to verify the two proposed methods. The maximum position errors and the time of each experiment are shown in Figure 13. Experiments 1–5 are in the quasi-static state while experiments 6–10 are in the swing state. Compared with method 1, the position accuracy based on the proposed method 2 can be improved up to 46.30% in the quasi-static state and 47.41% in the swing stare. Besides, compared with method 2, the position accuracy based on the proposed method 3 can be improved up to 17.24% in the quasi-static state and 17.66% in the swing stare. It is because the proposed method 2 makes the regression without using the piecewise function and considering the temperature gradient, that it plays a key role in describing the temperature field of single-axis RINS. In addition, the proposed method 3 introduces the coupled items of the temperature variables, which can reflect the complex temperature field thoroughly.

#### 5.2.2. Moving Vehicle Experiments Results

**Figure 14:**
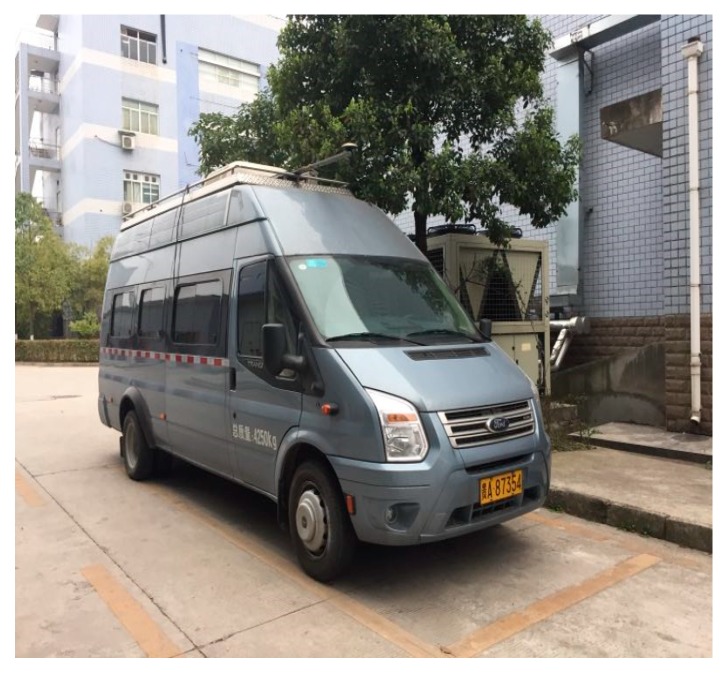
The vehicle used in the navigation experiment.

In order to verify the proposed calibration methods in practice, the moving vehicle experiments are carried out three times using the three calibration results. The vehicle used in the experiment is shown in Figure 14, which is a human-operated automobile equipped with a GPS receiver, a single-axis RINS and a computer for data visualization. The single-axis RINS is installed inside the vehicle and cold starts in the route including movements of uphill, downhill, turning, acceleration, and deceleration within 1.9 h.

**Figure 15:**
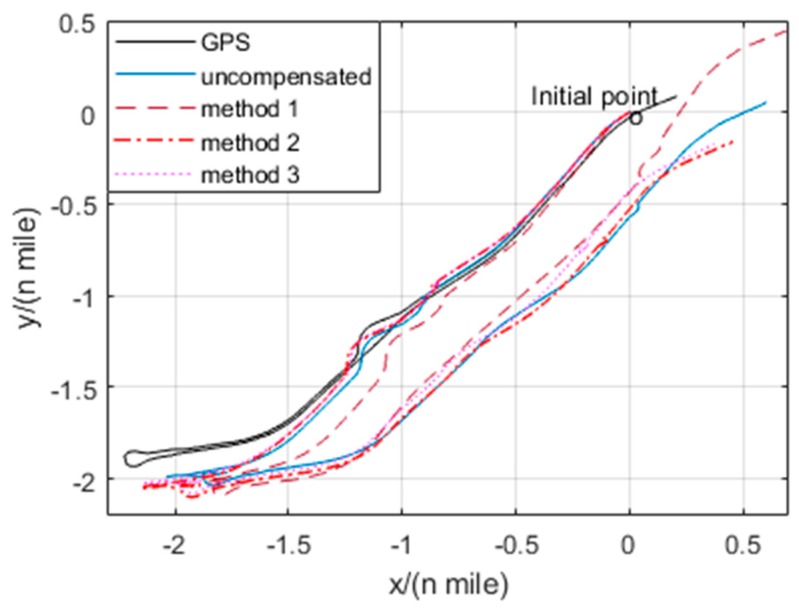
Trajectories of the navigation experiment.

**Figure 16:**
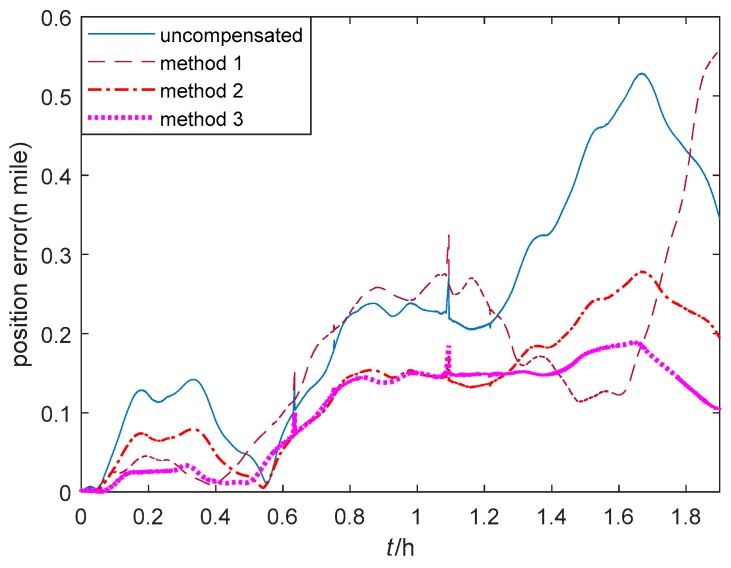
Position errors of the vehicle navigation experiment.

The trajectories of GPS output and the single-axis RINS navigation results based on the thermal calibration coefficients of method 1–3 are shown in Figure 15. The position errors of single-axis RINS based on three methods are compared with the GPS output as shown in Figure 16. It is shown that the navigation accuracy of method 3 is the highest, while that of method 1 is the lowest. As the temperature gradient and the coupled items of temperature variables are introduced to the thermal calibration model, and the IMU biases caused by the temperature field of single-axis RINS are fully compensated, this results in the changeless IMU biases in one rotation cycle of the torque motor. Hence, the navigation accuracy based on the proposed method 3 is improved by modulating the changeless IMU biases.

In order to verify the proposed methods thoroughly, two more vehicle navigation experiments are performed, and the maximum position errors of all three experiments are shown in Table 10. It is shown that the position error of method 3 can be decreased by 65.11% maximally compared to that of method 1. Besides, the results of three moving vehicle experiments show that the maximum position error can be reduced by 56% on average. Compared with the lab experiments, the improvements of the navigation accuracy in the vehicle experiments are even better, because the temperature change is more significant and the temperature gradient is more obvious. Hence, the proposed thermal calibration methods are superior to the traditional method in describing the temperature field of the work environment and improving the position precision.

## 6. Conclusions

This paper presents a study on the thermal calibration method based on multiple regression and BP neural networks for single-axis RINS. The effects of the change of IMU biases caused by the temperature are analyzed, and it is proven that the change of IMU biases should be considered in order to improve the position accuracy of single-axis RINS. A thermal calibration model is established with the temperature variables including the temperature, the temperature change rate and the temperature gradient, and the regression method is designed based on the relationship between the order of the thermal model and the RMS of calibration errors. To describe the complex temperature field thoroughly, the BP neural network with consideration of the coupled items between the temperature variables are introduced, and in addition, the number of neurons in the hidden layer are analyzed to improve the accuracy of estimation with the least computational complexity. Finally, experiments are conducted to test the performance of the two proposed methods. The results of navigation experiments in lab and field based on the traditional thermal calibration, the multiple regression method and BP neural network are compared. It is shown that the IMU outputs are continued without step-like appearance for the two proposed methods, and the RMS of calibration errors are less than that in the traditional thermal calibration method, because the temperature gradient and the coupled temperature items are considered in the model. It is concluded that the proposed thermal calibration methods can compensate the change of biases caused by temperature, which leads to the improvement in position accuracy of single-axis RINS.

## Figures and Tables

**Table 1:** The definition of frames.

Symbol	Frames
*i*	The orthogonal inertial frame
*n*	The orthogonal navigation frame directs east-north-up(ENU)
*e*	The earth-fixed frame
*r*	The rotation frame
*b*	The body frame

**Table 2:** The performance of thermal calibration equipment.

**Turntable**	
Principal and tilting axis rotation range	Continuous infinite
Principal and tilting axis angular position accuracy	±5′
**Thermal chamber**	
Temperature range	−50 °C~100 °C
Temperature change rate	±0.1~± 5 °C/min linear
**Temperature sensors**	
Temperature range	−50 °C~200 °C
Temperature measurement accuracy	0.01 °C

**Table 3:** The temperature points set by the thermal chamber.

Symbol	*T*_1_	*T*_2_	*T*_3_	*T*_4_	*T*_5_	*T*_6_	*T*_7_
**Temperature (°C)**	0	7	14	21	28	35	42

**Table 4:** The thermal calibration results of *x*-axis IMU based on method (1).

**Symbol**	**Temperature Range (°C)**						
	***T_Ax_* ≤ 4**	**4 < *T_Ax_* ≤ 10**	**10 < *T_Ax_* ≤ 17**	**17 < *T_Ax_* ≤ 23**	**23 < *T_Ax_* ≤ 30**	**30 < *T_Ax_* ≤ 37**	***T_Ax_* ≥ 37**
$A_{0x}$	7071.18	7243.84	7607.06	8138.59	9129.55	8919.01	12,730.53
$A_{T1x}$	−7.09	−66.89	−112.95	−145.75	−217.33	−148.79	−388.56
$A_{T2x}$	3.52	7.41	7.41	6.36	7.43	3.83	8.73
$A_{T3x}$	−0.32	−0.26	−0.16	−0.0915	−0.0842	−0.0324	−0.0650
$A_{\dot{T}1x}$	196.73	69.73	62.25	93.10	104.29	37.32	−31.43
$A_{\dot{T}2x}$	−153.01	62.48	479.76	521.83	411.17	476.53	544.07
$A_{\dot{T}3x}$	389.25	273.51	−129.38	−188.77	−111.36	−98.80	−101.98
**Symbol**	**Temperature Range (°C)**						
	*T_Gx_* ≤ 3	3 < *T_Gx_* ≤ 9	9 < *T_Gx_* ≤ 16	16 < *T_Gx_* ≤ 23	23 < *T_Gx_* ≤ 29	29 < *T_Gx_* ≤ 36	*T_Gx_* ≥ 36
$G_{0x}$	7367.37	7370.21	7363.00	7366.31	7370.28	7374.67	7355.78
$G_{T1x}$	0.468	−0.478	0.322	0.115	−0.0574	−0.209	0.316
$G_{\dot{T}1x}$	34.99	5.47	36.58	24.21	36.96	24.13	36.03

**Table 5:** The relationship between the model in method 2 and the RMS of calibration error.

Accelerometers Model	*RMS* (Pulse)			Gyroscopes Model	*RMS* (Pulse)		
	*Ax*	*Ay*	*Az*		*Gx*	*Gy*	*Gz*
*P* = 1, *q* = 1, *s* = 1	35.61	36.39	41.85	*P* = 1, *q* = 1, *s* = 1	24.36	22.06	14.10
*P* = 2, *q* = 2, *s* = 2	34.41	34.79	41.68	*P* = 2, *q* = 2, *s* = 2	24.36	22.05	14.10
*P* = 3, *q* = 3, *s* = 3	34.00	32.99	39.37	*P* = 3, *q* = 3, *s* = 3	24.36	22.04	14.09
*P* = 4, *q* = 4, *s* = 4	33.93	32.93	39.18	*P* = 4, *q* = 4, *s* = 4	24.35	22.04	14.08

**Table 6:** The thermal calibration results based on method 2.

Symbol	Value	Symbol	Value	Symbol	Value	Symbol	Value	Symbol	Value
$A_{0x}$	7411.39	$A_{T1x}$	−8.52	$A_{T2x}$	0.180	$A_{T3x}$	−1.84e-3	$A_{\dot{T}1x}$	92.22
$A_{\dot{T}2x}$	256.82	$A_{\dot{T}3x}$	−148.15	$A_{\Delta T1x}$	−533.16	$A_{\Delta T2x}$	280.29	$A_{\Delta T3x}$	−45.09
$A_{0y}$	7051.03	$A_{T1y}$	3.41	$A_{T2y}$	0.0575	$A_{T3y}$	−1.73e-3	$A_{\dot{T}1y}$	94.10
$A_{\dot{T}2y}$	118.68	$A_{\dot{T}3y}$	−18.01	$A_{\Delta T1y}$	−407.06	$A_{\Delta T2y}$	191.04	$A_{\Delta T3y}$	−27.73
$A_{0z}$	465,231.31	$A_{T1z}$	−10.31	$A_{T2z}$	0.122	$A_{T2z}$	−2.61e-4	$A_{\dot{T}1z}$	−128.82
$A_{\dot{T}2z}$	−23.17	$A_{\dot{T}3z}$	−1.41	$A_{\Delta T1z}$	−290.31	$A_{\Delta T2z}$	163.01	$A_{\Delta T3z}$	−25.43
$G_{0x}$	7361.77	$G_{T1x}$	−0.0175	$G_{\dot{T}1x}$	40.48	$G_{\Delta T1x}$	3.87		
$G_{0y}$	−3.56	$G_{T1y}$	−0.0158	$G_{\dot{T}1y}$	14.85	$G_{\Delta T1y}$	0.592		
$G_{0z}$	3617.88	$G_{T1z}$	−0.0419	$G_{\dot{T}1z}$	4.82	$G_{\Delta T1z}$	0.397		

**Table 7:** The relationships between the model in method 3 and the RMS of calibration error.

Accelerometers Neurons Number	*RMS* (Pulse)			Gyroscopes Neurons Number	*RMS* (Pulse)		
	*Ax*	*Ay*	*Az*		*Gx*	*Gy*	*Gz*
*n* = 4	32.91	32.22	38.97	*n* = 1	24.85	22.14	14.09
*n* = 5	32.86	32.45	38.82	*n* = 2	24.21	22.02	14.07
*n* = 6	32.61	32.20	38.68	*n* = 3	24.14	22.00	14.04
*n* = 7	32.60	32.19	38.67	*n* = 4	24.13	22.02	14.03
*n* = 8	32.59	32.21	38.67	*n* = 5	24.14	22.01	14.04

**Table 8:** The calibration results of x-axis IMU based on method 3.

Accelerometers						Gyroscopes	
Symbol	Value	Symbol	Value	Symbol	Value	Symbol	Value
*w*_11_	−1.36	*w*_13_	8.01	*v*_1_	−0.0327	*w*_11_	−1.25
*w*_21_	−7.24	*w*_23_	1.16	*v*_2_	0.0927	*w*_21_	0.228
*w*_31_	−0.316	*w*_33_	0.562	*v*_3_	3.87	*w*_12_	0.414
*w*_41_	−0.137	*w*_43_	0.227	*v*_4_	−10.00	*w*_22_	0.355
*w*_51_	−2.038	*w*_53_	1.10	*v*_5_	0.632	*w*_13_	−0.588
*w*_61_	−17.37	*w*_63_	5.06	*v*_6_	0.116	*w*_23_	0.124
*w*_12_	−4.14	*b*_1_	6.55	*b*′	1.35	*b*_1_	1.40
*w*_22_	−0.540	*b*_2_	−1.01			*b*_2_	−1.54
*w*_32_	−0.153	*b*_3_	−0.356			*v*_1_	7.84 × 10^−3^
*w*_42_	−0.0919	*b*_4_	0.0561			*v*_2_	0.148
*w*_52_	−0.490	*b*_5_	1.56			*b*′	0.125
*w*_62_	−1.24	*b*_6_	−12.13				

**Table 9:** The quasi-static and swing condition of the turntable.

Turntable Condition	The Quasi-Static State	The Swing State	
	Amplitude (°)	Frequency (Hz)	Amplitude (°)
**Heading**	45	2	3
**Pitch**	0	2	3
**Roll**	0	8	5

**Table 10:** The maximum position errors of the vehicle navigation experiment.

Experiment Number	Method 1	Method 2	Method 3	The Decreased Percentage ^2^
Experiment 1	0.556 n mile ^1^	0.277 n mile	0.194 n mile	65.11%
Experiment 2	0.724 n mile	0.343 n mile	0.296 n mile	59.11%
Experiment 3	0.495 n mile	0.316 n mile	0.282 n mile	43.03%

^1^ 1 n mile (nautical mile) ≈ 1.852 km.^2^ The decreased percentage of maximum position error between method 3 and method 1.
